# Genome-wide profiling of sRNAs in the *Verticillium dahliae*-infected *Arabidopsis* roots

**DOI:** 10.1080/21501203.2018.1426062

**Published:** 2018-01-23

**Authors:** Yun Jin, Pan Zhao, Yuan-Yuan Fang, Feng Gao, Hui-Shan Guo, Jian-Hua Zhao

**Affiliations:** a State Key Laboratory of Plant Genomics, Institute of Microbiology, Chinese Academy of Sciences, BeijingChina; b College of Life Science, University of the Chinese Academy of Sciences, Beijing, China; c College of Agriculture, Shihezi University and Key Laboratory at Universities of Xinjiang Uygur Autonomous Region for Oasis Agricultural Pest Management and Plant Protection Resource Utilization, Shihezi, China

**Keywords:** RNAi, small RNA, miRNA, *Verticillium dahliae*, sRNA sequence

## Abstract

Small RNAs (sRNAs, including small interfering RNAs [siRNAs] and micro RNAs [miRNAs]) are key mediators of RNA silencing (or RNA interference), which play important roles in plant development and response to biotic and abiotic stimulation. Verticillium wilt is a plant vascular disease caused by the soil-borne fungal pathogens, such as *Verticillium dahliae*. We previously reported that *V. dahliae* infection increased two plant endogenous miRNAs that were exported to fungal cell to silence virulence genes. To investigate plant sRNAs in genome-wide response to *V. dahliae* infection, in this study, we constructed two sRNA libraries from *Arabidopsis* roots with and without *V. dahliae* infection, respectively. In total, 31 conserved miRNAs were found to be differentially expressed during the early stage of infection with *V. dahliae* using sRNA sequencing. Among these, the expression levels of miR160, miR164, miR166, miR167, miR390 and miR156h were confirmed by northern blot. Reverse transcription quantitative real time polymerase chain reaction results showed that the induction of miRNAs (miR160, miR164, miR166 and miR167) upon *V. dahliae* infection downregulated the expression of their targeted genes (*ARF10, NAC1, PHV* and *ARF6*), respectively. In addition, we identified specific phased siRNAs generated from distinct regions of two libraries. Profiling of these miRNAs and sRNAs lay the foundation for further understanding and utilising the host-induced gene silencing strategy to control plant vascular pathogens.

## Introduction

Many plant pathogens cause severe crop disease and significant yield loss every year (Klosterman et al. 2009). As a representative of soil-borne fungal pathogens and the causing agent of vascular wilt disease, *Verticillium dahliae* poses a threat to over 400 plant species worldwide, including economically important crops such as cotton, potato and sunflowers (Klosterman et al. 2009). Due to the long-term survival in the soil and colonisation inside host plants, *V. dahliae* cannot be controlled by fungicides (Zhao et al. 2014; Zhang et al. 2016a).

The plant immunity triggered by perception of pathogen-associated molecular patterns (PAMPs) or microbe-associated molecular patterns is the first layer of plant defence, and able to restrict vast majority of potential pathogens encountered by plants (Boller and He 2009; Monaghan and Zipfel 2012; Wong et al. 2014). To successfully colonise host plants, pathogens secrete effectors, which are often small and cysteine-containing proteins, to target key components of the defence system and effectively subvert PAMP-triggered immunity (Boller and He 2009; Monaghan and Zipfel 2012; Pumplin and Voinnet 2013; Wong et al. 2014). The mechanism of how effectors modulate plant immunity is largely unclear, especially in RNA interference (RNAi)-involved pathways which usually play pivotal roles in both plant immunity as well as pathogen infection (Bozkurt et al. 2012; Qiao et al. 2013). Previous study identified *Pseudomonas syringae* effectors that suppress transcriptional activation of PAMP-responsive micro RNAs (miRNAs) or miRNA biogenesis, stability or activity, and *Arabidopsis* miRNA-deficient mutants partly restored growth of *P. syringae* secretion-defectivemutant (Navarro et al. 2008). Hundreds of predicted effector genes from oomycete genomes reflect more complex defence–counterdefence crosstalk than that in plant–bacteria interactions (Thines and Kamoun 2010; Qiao et al. 2013). Recent results showed that two effectors from the oomycete pathogen *Phytophthora sojae* can suppress plant RNA silencing (or RNAi) by inhibiting the biogenesis of small RNAs (sRNAs) (Qiao et al. 2013). These findings suggest that modulation or suppression of host RNAi pathways is a common strategy used by pathogens for host colonisation.

RNAi is an evolutionary conserved and sequence-specific mechanism that induces mRNA degradation or inhibits translation at either the post-transcriptional level or the transcriptional level (Duan et al. 2012; Bologna and Voinnet 2014; Zhao et al. 2016b). sRNAs are the key mediators of RNAi and are produced from double-stranded RNA (dsRNA) by an RNase III-type enzyme(Bologna and Voinnet 2014). Based on their origin and formation, these sRNAs are mainly classified into two major types: the small interfering RNAs (siRNAs) and the miRNAs (Bologna and Voinnet 2014). RNAi is involved in a wide range of biological processes and plays a major role in maintaining the balance between investment in growth and development and defence against biotic and abiotic stress (Carrington and Ambros 2003; Lai 2003; Matzke and Birchler 2005; Zhao et al. 2016b). Many studies disclose that one important example is its role as an inducible plant defence pathway that targets or inactivates invading nucleic acids from viruses (Mlotshwa et al. 2008; Zhao et al. 2016b). Apart from viral defence, evidence accumulates for RNAi involving in interactions with other pathogen types (Pumplin and Voinnet 2013). Previous study identified that some *Botrytis cinerea* sRNAs can hijack host RNAi by binding to host Argonaute 1 (AGO1) to silence *Arabidopsis* and tomato genes involved in immunity (Weiberg et al. 2013). In *Arabidopsis* and tomato, expressing sRNAs that target *B. cinerea DCL* gene attenuated fungal pathogenicity and growth (Wang et al. 2016). RNAi-deficient *Arabidopsis* mutants showed affected defence against *Verticillium* and *Fusarium*, but not other fungal pathogens including *Alternaria brassicicola, B. cinerea and Plectosphaerella cucumerina* (Ellendorff et al. 2009).

The host-induced gene silencing (HIGS) has emerged in application to control plant pathogens, including fungi and oomycetes (Nowara et al. 2010; Tinoco et al. 2010; Jahan et al. 2015; Zhang et al. 2016a). Recently, we generated transgenic cotton plants expressing an RNAi construct targeting *V. dahliae* hydrophobin gene (designed as *VdH1i*), and demonstrated the efficiency and effectiveness of producing *VdH1i*-derived siRNAs in transgenic cotton plants to confer resistance against *V. dahliae* infection (Zhang et al. 2016a). Moreover, we discovered that during cotton–*V. dahliae* interaction, large amount of miR166 and miR159 were transmitted from cotton plants to *V. dahliae* hyphae and targeted fungal virulence genes for silencing (Zhang T, Zhao et al. 2016b). These results motivated us to profile plant siRNAs in response to *V. dahliae* infection. In this study, we profiled siRNAs in the model plant *Arabidopsis thaliana* (Col-0) responding to *V. dahliae* infection by Illumina sequencing. We aim to investigate miRNAs and other small non-coding RNAs in the *V. dahlia*-infected roots. By analysing sequencing data and Northern blot, we identified specific plant miRNAs and siRNAs that were differentially accumulated at the early stage of *V. dahliae* infection.

## Results

### sRNA profiling in *Arabidopsis* roots infected by *V. dahliae*

To understand the role of *Arabidopsis* sRNAs during *V. dahliae* (V592) infection, we profiled the expression of sRNAs in the *Arabidopsis* that was inoculated with V592 strain at 5 days post inoculation (dpi). At this time point, the hyphae reached the vascular intercellular tissue through the cortical cells, and a hyphal net within the xylem vessels was observed (Zhao et al. 2014). We hypothesise that the sRNAs were altered at this early stage of infection and are involved in regulating defence response.

**Figure 1.**
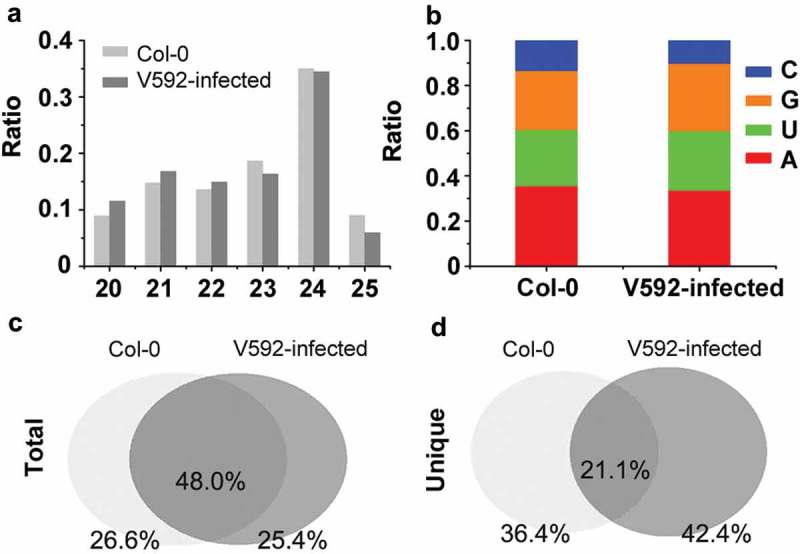
The profiles of sRNA in Col-0 and V592-infected roots. (a) The length distribution of sRNAs from roots in Col-0 and V592-infected plants. (b) The first nucleotide preference of sRNAs from roots in the Col-0 and V592-infected plants. (c) and (d) The overlap reads of total sRNAs (c) and unique sRNAs (d) between Col-0 and V592-infected root.

To verify this hypothesis, we extracted total RNA from the roots of Col-0 with and without V592-inoculation. Two sRNA libraries were constructed and subjected to Illumina sequencing. After trimming the adapter sequences and removal of low quality reads, more than 10 million clean reads from each library were obtained (Table S1). Approximately 80% of the clean reads were mapped perfectly to *Arabidopsis* genome (Table S1). sRNAs with length between 20 and 25 nt were included in our analysis. Previous studies reported that *Arabidopsis* sRNAs are sorted into distinct AGO complexes depending on the length and 5’ first nucleotide of sRNAs, which has important implications for the functions of miRNAs and siRNAs (Mi et al. 2008; Takeda et al. 2008). Therefore, we detected the length and 5’ first nucleotide of sRNAs in the two libraries respectively. There were no significant differences in the percentages of reads in different length (Figure 1(a)) nor the 5’ first nucleotide of sRNAs (Figure 1(b)) between the two libraries generated from Col-0 and V592-infected Col-0. Next, we assessed the features of the perfectly matched siRNAs duplexes and cis-NAT (cis-natural antisense transcripts) siRNAs in the two libraries. We found that the siRNAs of 24-nt class with 1A dominated the siRNAs duplexes in the two libraries (Table S2). Very little siRNAs generating from cis-NATs were identified in the two libraries, which is consistent with previous research work on root siRNA sequence analysis (Wang et al. 2011).

The population structure of endogenous siRNAs from V592-infected and uninfected Col-0 plants was similar, making us to further compare the sequence of siRNAs in the two libraries. The result shows that sample-specific siRNA sequences accounted for about 26% of total sequence reads in each library (Figure 1(c)), whereas only about 20% siRNA sequences were shared by both libraries (Figure 1(d)). These observations highlighted major differences of two siRNA libraries, suggesting RNAi regulation underlying plants in response to *V. dahliae*.

### Identification of differentially accumulated miRNAs in col-0 and v592-infected plants

To disclose the role of sRNAs against *V. dahliae* infection, we first compared the expression level of conserved miRNAs between V592-infected and uninfected Col-0 plants. miRBase database (release 21) recruits 472 annotated miRNA in *Arabidopsis*, including 349 unique sequences. To simplify the sequence data, all identical sequences in miRBase database were grouped and associated with one annotation. Only miRNAs with reads more than 50 in one of the two libraries were selected for further analysis, and in total there were 73 miRNAs satisfying this criterion. Therefore, we compared the normalised number of reads in per million (RPM) of these 73 miRNAs.

**Figure 2.**
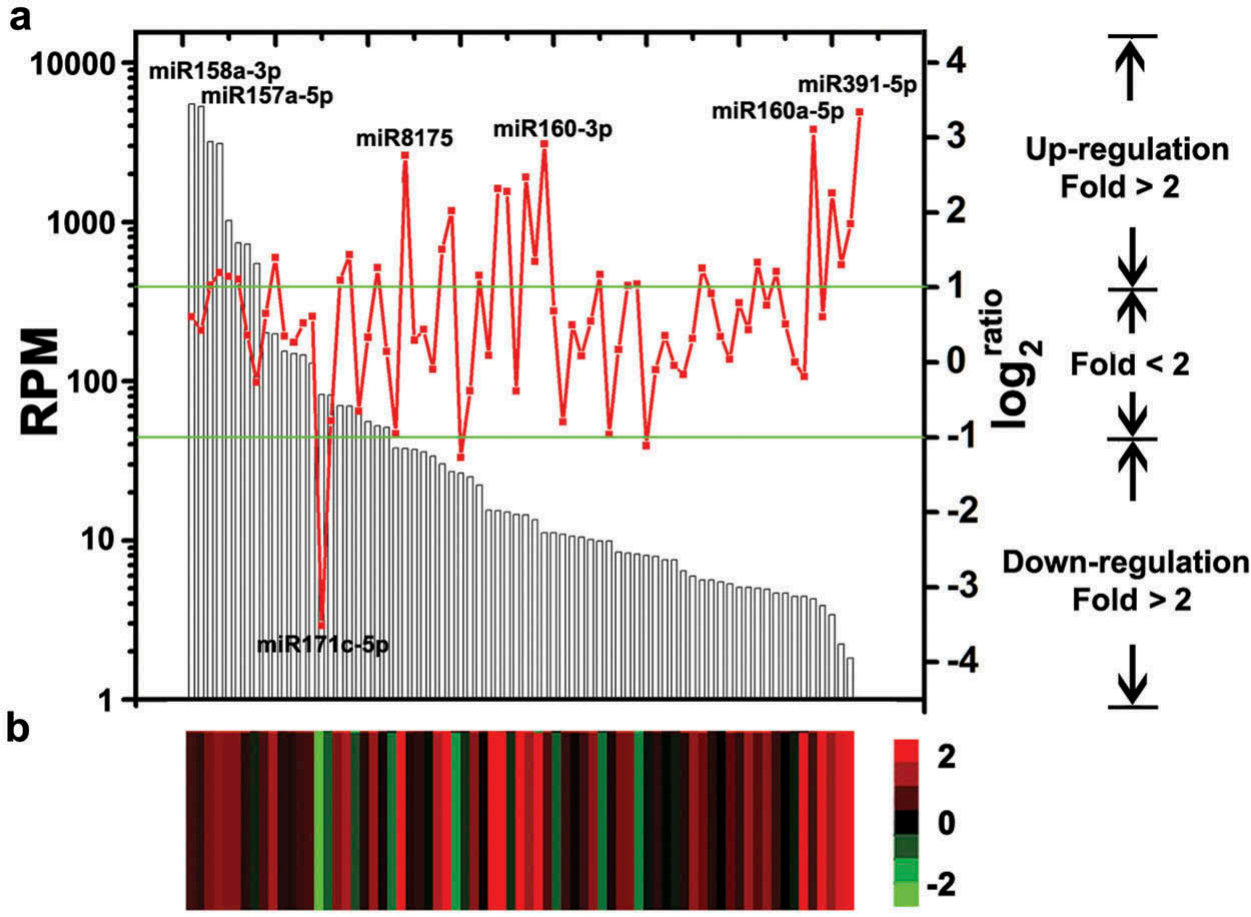
The miRNAs expression in Col-0 and V592-infected roots. (a) The columns show the 73 analysed miRNAs expression in Col-0 corresponding to the left *y*-axis. The broken line shows the change fold of miRNAs expression between the Col-0 and V592-infected (corresponding to the right *y*-axis). Twenty-eight miRNAs above the upper line and three below the lower line represent the significant increases (fold > 2) or decreases. (b) Heatmap shows the differential expression of miRNAs in Col-0 and V592-infected roots. Log2 transformations of the expression fold changes (V592-infected vs. Col-0) are corresponding to broken line.

Statistical analysis of miRNA abundance between libraries identified 31 miRNAs (42.5% of the 73 analysed miRNAs) that were differentially expressed (*P*-value < 0.05, fold > 2) between V592-infected or uninfected Col-0 plants (Figure 2, Table S3). Most of the miRNAs (28 out of 31), including miR159 and miR166 that were previously found to be induced by V592 infection (Zhang T, Zhao et al. 2016b), were induced in the V592-infected plants, except for miR171c-5p, miR168a-3p and miR156j (Figure 2, Table S3). We previously found that miR167 was not induced by V592 infection in either cotton or *Arabidopsis* roots (Zhang T, Zhao et al. 2016b). However, miR167 was found to be V592-induced at 5 dpi (Figure 2, Table S3), suggesting it was dynamic in early and later response upon infection. In the total data set, the miR158a-3p and miR157a-5p had a dominant number of reads and more than 5000 RPM in two libraries. However, no significantly differential expression of these two miRNAs was found between V592-infected or uninfected Col-0 plants. Five miRNAs, miR171c-5p, miR391-5p, miR160a-5p, miR8175 and miR160a-3p are most sensitive to V592 infection (Figure 2(a), Table S3). The expression of miR171c-5p, miR8175 and miR160a-3p were moderate, meanwhile, the miR391-5p and miR160a-5p showed low abundance of reads compared to other miRNAs (Figure 2(a)) in Col-0 plants. These results showed that the miRNA expressions in response to V592 infection did not necessarily correlate to the reads abundance of the specific miRNA (Figure 2(a)). Also, we could not preclude that miRNAs with low reads abundance and with no differential expression might have roles in response to *V. dahliae* infection in *Arabidopsis* roots.

**Figure 3.**
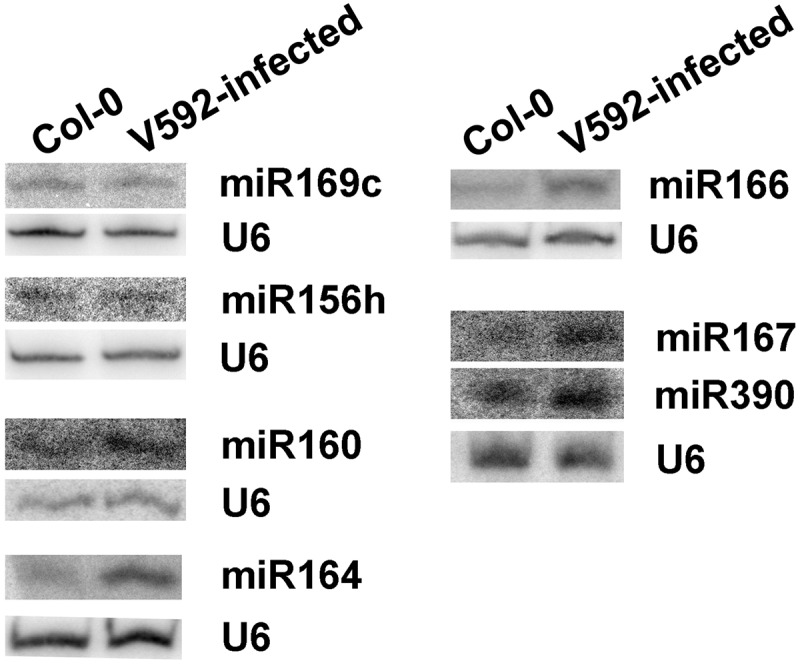
Northern blots confirm the altered miRNAs expression in Col-0 and V592-infected roots. U6 served as the loading control.

To confirm the altered accumulation of miRNAs, northern blot analysis was used to detect miRNA expression in V592-infected and uninfected Col-0 roots. The miRNAs including miR160, miR164, miR166, miR167 and miR390, which have different accumulation in two libraries and induced by V592 infection were selected. Two unchanged miRNAs, miR156h which has a different sequence from other miRNAs in miR156 family and miR169c, were also selected. The results generated by northern blot were consistent with the sRNAs in illumina sequencing results, although the fold changes and expression levels were sometimes different (Figure 3).

### Comparative expression of miRNA-targeted genes

**Figure 4.**
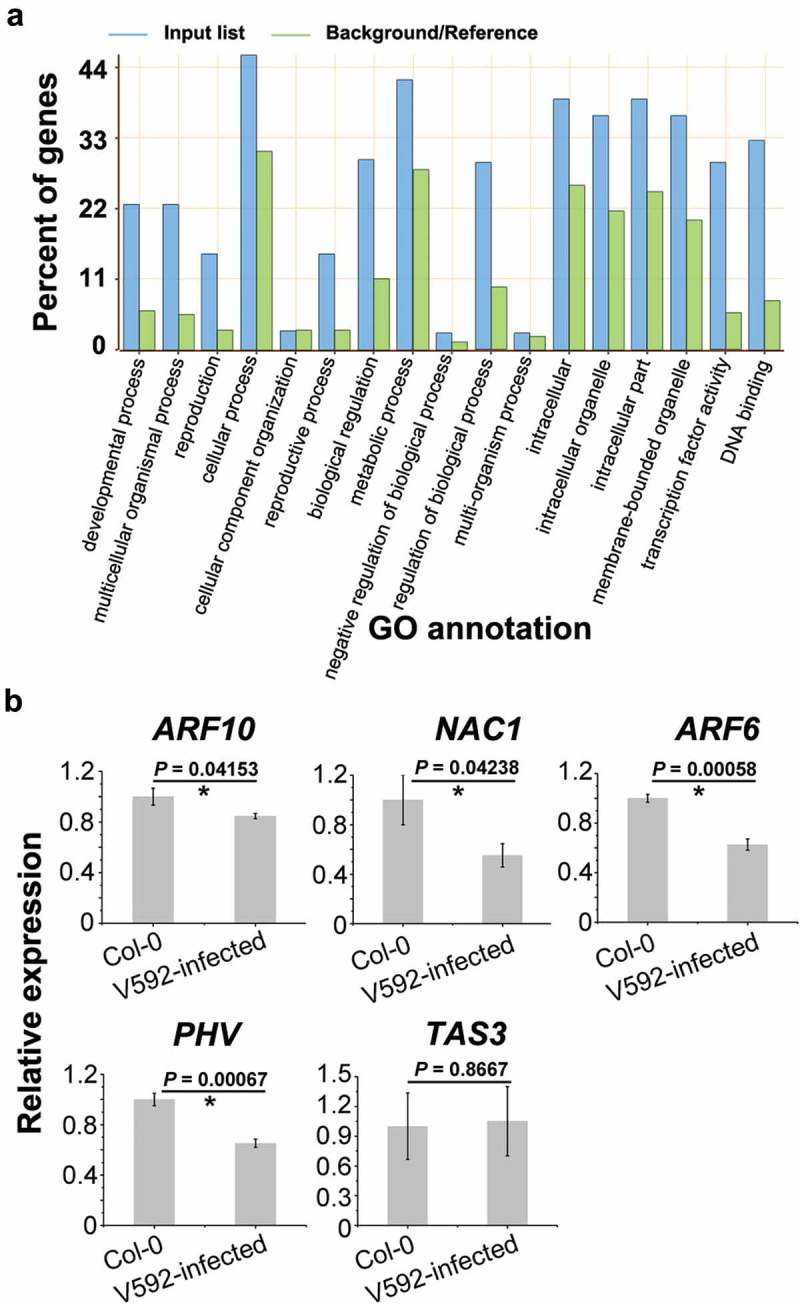
The function and expression of altered miRNAs targeted genes. (a) The target genes of altered miRNAs were categorised into different GO. (b) The expression of genes targeted by miR160 (*ARF10*), miR164 (*NAC1*), miR166 (*PHV*), miR167 (*ARF6*) and miR390 (*TAS3*).

To assess the influence of the miRNAs on putative targets, we analysed the correlation between miRNAs and predicted *Arabidopsis* gene targets. The expression of target genes should be negatively correlated with miRNA expression, since miRNA triggers degradation of target mRNA (Zhang et al. 2010). The result of GO analysis shows that miRNA-targeted genes induced in V592-infected plants were involved in extensive pathways (Figure 4(a), Table S4), which is consistent with the complicated role of miRNAs in plant developments and responses to biotic or abiotic stimulation.

Five genes, *ARF10, NAC1, ARF6, PHV* and *TAS3*, targets of miR160, miR164, miR167, miR166 and miR390, respectively, were investigated for transcript accumulation through quantitative RT-PCR (RT-qPCR) analysis (Figure 4(b)). Among these genes, *ARF10, NAC1, TAS3* and *ARF6* are known to be involved in auxin signals transduction (Chandler 2016). *PHV* targeted by miR166 is functional in shoot and root apical meristems (Grigg et al. 2009). Opposed to miRNAs induced by V592-infection, the expression of *ARF10, NAC1, ARF6* and *PHV* was significantly reduced (Figure 4(b)) (*P*-value < 0.05, *t*-test). These results suggested that auxin response plays a role in plants responding to V592-infection with complex regulatory. Interestingly, we did not find significant difference in expression of *TAS3*, which is target of miR390, between two samples (*P*-value > 0.05, *t*-test). This result prompted us to detect the accumulation of *TAS*-derived trans-acting siRNAs (ta-siRNAs) upon V592 infection.

### Genome-scale analysis of ta-siRNAs and phased siRNAs (phasiRNAs) in *Arabidopsis* during V592 infection

**Figure 5.**
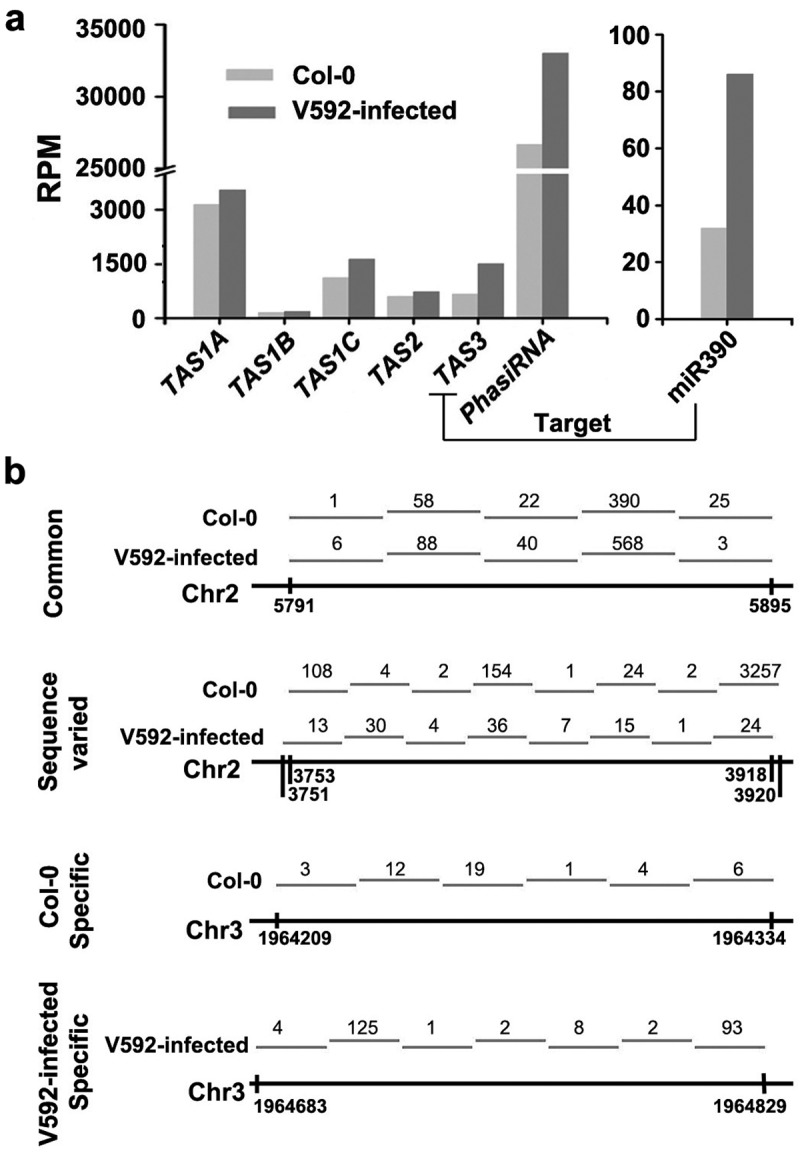
The distribution of ta-siRNAs and phasiRNAs in the Col-0 and V592-infected roots. (a) The accumulation of miR390, ta-siRNAs and phasiRNAs in the Col-0 and V592-infected roots. (b) The distribution of common or sample-specific phasiRNAs was in the Col-0 and V592-infected roots. Numbers of phasiRNAs cloned were indicated above the short lines represented the phasiRNAs in different regions.

Previous reports showed that ta-siRNAs are processed sequentially from *TAS* gene transcripts derived dsRNAs following cleavage by miRNAs (Wang et al. 2011; Wu et al. 2012). Since miR390 was induced but *TAS3* transcript was not changed in V592-infected plants compared to uninfected Col-0 plants, we assayed the ta-siRNAs derived from all the *TAS* gene transcripts. Although we did not find significant difference in the accumulation of total ta-siRNAs between two samples (Figure 5(a)) (*P*-value > 0.05, *t*-test), the ta-siRNAs generated from *TAS3* which targeted by miR390 were significantly increased in V592-infected Col-0 (*P*-value < 0.05, Fisher-test) (Figure 5(a)). The results showed that the expression level of miRNA was proportional to the ta-siRNAs that generated from the target *TAS3* gene (Figure 5(a)). The ta-siRNA generated from distinct targets of one miRNA at different levels may be due to the capacity of targets binding to the miRNA. Nevertheless, increase in *TAS3*-derived ta-siRNAs without reduction of *TAS3* transcript in V592-infected Col-0, suggests increase in *TAS3* transciption in response to V592 infection in keeping production of *TAS3*-derived ta-siRNAs, that in turn to target *ARF2, ARF3* and *ARF4* involving in auxin signal pathway (Fahlgren et al. 2006).

In addition to known ta-siRNAs, we also identified phasiRNAs in the genome-scale. Through analysis of the 21-nt siRNAs, we detected many phasiRNAs clusters in two libraries and the accumulation of phasiRNAs increased in V592-infected plants (*P* < 0.05, chi-square test) (Figure 5(a)). Next, we assayed the distribution of phasiRNAs in the genome. The results show that many phasiRNAs were generated from the same regions in the genome (Table S5), whereas some specific phasiRNAs were generated from distinct regions (Figure 5(b), Table S5). Moreover, phasiRNAs generated from the same genomic region have various sequences in the two libraries (Figure 5(b)). These data suggest that V592 infection also affected the production of phasiRNAs. Further investigation is however required to determine whether the different production of these phasiRNAs depended on miRNAs-guided cleavage as that of ta-siRNAs during *V. dahliae* infection.

## Discussion

In present study, we found specific miRNAs and siRNAs differentially accumulated in *V. dahliae*-infected and uninfected *Arabidopsis* roots. The expression of miR160, miR164, miR166, miR167 and miR390 were induced upon *V. dahliae* infection and validated by northern blot analysis, and their target genes except for *TAS3* showed down-regulation in V592-infected plant roots as anticipated.

In response to *V. dahliae* infection, the increased microRNAs included miR160, miR164, miR167 and miR390 that are involved in regulating auxin signals, which play critical roles in almost all aspects of plant developments such as organogenesis, vascular tissue differentiation, apical dominance and root initiation, and in responses to the environmental changes (Chandler 2016; Li et al. 2016). Many previous results showed that RNAi pathway is involved in auxin signalling. A large number of candidate genes that are potentially regulated by auxins, including the *ARF* and *IAA* gene family in *Arabidopsis*, are targets of miRNAs (Szemenyei et al. 2008; Chandler 2016). Previously, we demonstrated that auxin-dependent induction of miR847 is involved in regulating meristematic competence by targeting the mRNA of *IAA28* to up-regulate auxin signalling (Wang and Guo 2015). Furthermore, many *ARF* genes are directly regulated by at least one miRNA in response to biotic or abiotic stress, such as *ARF6* and *ARF8* are targeted by miR167 while *ARF10, ARF16* and *ARF17* are targeted by miR160 (Chandler 2016; Li et al. 2016). *ARF2, ARF3* and *ARF4* are also targeted by a ta-siRNA, encoded by the *TAS3* locus, which is targeted by miR390 (Chandler 2016; Li et al. 2016).

In plant–pathogen interactions, auxin also involves in the regulation of changes in different growth processes to maintain the balance between defence and beneficial growth responses (Ludwig-Muller 2015). Previous study showed that *Arabidopsis* mutants defective in different aspects of the auxin pathway are more susceptible than wild-type plants to *A*. *brassicicola* (Qi et al. 2012). Many soil-borne pathogens infect the roots through auxin-rich regions (Kazan and Manners 2009), such as the root-infection fungus *Fusarium oxysporum*, which infection leads to alteration in auxin homeostasis (Kidd et al. 2011). *Arabidopsis* mutants defective in auxin signalling show increased resistance to *F. oxysporum* (Kidd et al. 2011). These results show that auxin pathways contribute to the defence network in plants.

Our analysis showed the increase in *TAS3*-derived ta-siRNAs without reduction of *TAS3* transcript in V592-infected Col-0. It was likely contradictory. However, this finding suggests that increase in *TAS3* transcription was consistent with the increased miR390 in response to V592 infection, presumably resulting in continuous production of *TAS3*-derived ta-siRNAs to target *ARF2, ARF3* and *ARF4* that affect developmental timing and patterning in *Arabidopsis* (Fahlgren et al. 2006). How induced *TAS3* transcript in response to *V. dahliae* infection requires further investigation.

The miR164 can regulate multiple aspects of plant growth and development by targeting different mRNAs (Guo et al. 2005). In inflorescences, miR164 targets *CUC1/2* to regulate the establishment and maintenance of the shoot apical and axillary meristems and floral boundary formation (Laufs et al. 2004; Mallory et al. 2004; Baker et al. 2005), whereas in root miR164 targets *NAC1* to downregulate auxin signals for lateral root development (Guo et al. 2005). Our sRNA sequencing and northern blot results confirmed that miR164 can be induced by V592-infection. Furthermore, RT-qPCR results showed that the expression of miR164 target *NAC1* gene was inversely proportional to miR164 expression (Figures 3 and 4). *NAC1* is a transcription factor and specifically expressed in *Arabidopsis* roots (Xie et al. 2000). Since *V. dahliae* is a soil-borne fungus and obligate in root infection, involvement of miR164 and *NAC1* in regulation of *V. dahliae* infection is worthy of further investigation.

sRNAs have also been widely studied in regulating genes for immunity (Carrington and Ambros 2003). Research of HIGS showed that host sRNAs generated by artificial transgenic expression can translocate into colonised pathogen cells to turn down virulence gene expression (Wang et al. 2015). In the last few years, HIGS had proven to efficiently control pests, nematodes, filamentous pathogens and parasitic plants (Wang et al. 2016). Our latest study described that transgenic cotton plants expressing artificial siRNAs specifically targeting *VdH1i* gain significant resistance against vascular wilt disease (Zhang et al. 2016a). In this study, we found that responding to *V. dahliae* infection, some host phasiRNAs were highly accumulated whereas others were restraint. It is interesting to investigate whether phasiRNAs were exported into hyphae to target fungal genes, similar to miR166, which was found to be exported to hyphae to specifically silence fungal virulence genes as our recent report (Zhang T, Zhao et al. 2016b). On the other side, pathogens also produce sRNAs. Similar as effector proteins, pathogen sRNAs were also found to be delivered into host cells and suppress host immunity (Weiberg et al. 2013). For instance, 3 out of 70 *B. cinerea* sRNAs have been demonstrated experimentally to hijack plant RNAi machinery with sorting into host AGO1 protein, and favour successful *B. cinerea* infection (Weiberg et al. 2013; Wang et al. 2015). *Arabidopsis ago1* mutant plant was less susceptible to *B. cinerea*, since sRNAs were no longer functional in guiding the host gene silencing (Weiberg et al. 2013; Wang et al. 2015). The *ago1* mutant plant showed enhanced resistant against *V. dahilae*, implicating that *V. dahilae* has evolved a similar strategy as *B. cinerea* to suppress host immunity (Ellendorff et al. 2009; Wang et al. 2015). We also obtained large amount of *V. dahliae*-derived sRNAs and further investigation should be carried out to determine whether *V. dahliae*-derived sRNAs were transferred into *Arabidopsis* cells. Nevertheless, all these findings reveal the natural cross-kingdom RNAi during plant-microbe interactions, and it is of important and worth to develop effective means of controlling the devastating Verticillium wilt disease based on the natural efficient uptake of host RNAi triggers by pathogenic fungi.

## Materials and methods

### Plant material and root dip-inoculation assay

*Arabidopsis thaliana* ecotypes Columbia (Col-0) were used in the infection assays. A virulent defoliating *V. dahliae* isolate, V592, from cotton originated in Xinjiang, China, was used in this study.

The Col-0 seeds were surface sterilised and stored in wet conditions for 3 days at 4°C. The sterilised seeds were sown on Murashige and Skoog medium (Duchefa Biochemie, the Netherlands) solidified. After 10 days, the seedlings were transferred to a liquid culture vessel filled with one-fourth nutrient solution without organotrophy (Zhao et al. 2014), and were grown in a greenhouse at 25°C with 16h-light/8h-dark cycles. For *V. dahlia* inoculations, 10-day-old Col-0 plants were incubated in the 5 × 10^6^ conidia/ml with sterile distilled water for 10 min. Control plants were dipped in sterilised distilled water for the same amount of time. The plants were then placed into a greenhouse under the above-mentioned conditions for the observations of the development of symptoms for 5 days.

### sRNA library construction

The *Arabidopsis* roots were harvest for sRNA library construction. The total RNAs were extracted from the Col-0 and V592-infected plant roots using TRIzol reagent (Invitrogen, USA) according to the manufacturer’s instructions. sRNAs library construction and sRNA sequencing were carried out by BGI (http://www.bgitechsolutions.com/).

### Bioinformatics analysis of sequencing data

After trimming adaptor sequences, all clean reads were mapped to the genome of *Arabidopsis* (TAIR9) using Short Oligonucleogide Analysis Package (SOAP) alignment software (Li et al. 2009). sRNAs with lengths between 20 and 25 nt, which were perfectly matched to *Arabidopsis* genomic sequences, were included in our analysis.

Known miRNA in *Arabidopsis* were downloaded from miRBase release 21 (Griffiths-Jones et al. 2008). Perl scripts were used to search these miRNA sequences from our sRNA libraries. The targets of miRNA in *Arabidopsis* were downloaded from Plant MicroRNA Database (Zhang et al. 2010). The function of these targets was annotated by AgriGO (Du et al. 2010).

sRNA abundance was normalised into RPM to compare sRNA expression between libraries.

The genome loci of cis-NAT were referred to the previous study results (Jin et al. 2008; Zhang et al. 2012). Blast was used to match the sRNA to these loci.

siRNAs with 21 nt and mapped consecutively to the genome of *Arabidopsis* were selected. We defined phasiRNA as those having no less than three siRNAs in a series, and Perl scripts were used to predict the phasiRNAs and calculate the expression level. According to SOAP mapped results, we searched the phasiRNAs depended on the start and end site on the genome.

### miRNA northern blot

For sRNA gel blosts, 30 μg of total RNA was separated by electrophoresis on 17% PAGE gels and electrically transferred to nylon N+ membrane. [α-^32^P]-UTP-labelled gene-specific transcript sequences were used (New England Biolabs, U.K.). All probe sequences are listed in Table S6. The blots were hybridised in hybridisation buffer containing 50% formamide, 7% sodium dodecyl sulfate, 50 mM sodium phosphate buffer (pH 7.2), 5×Denhardt’s solution and 0.3 M NaCl at 40°C overnight. After two washes with 2×standard saline citrate/0.2% SDS, the blots were immediately exposed to a storage phosphor screen for 10 h.

### Quantitative real-time PCR

To identify the expression of miRNA targets, total RNA was extracted from roots using TRIzol reagent (Invitrogen, USA), digested genomic DNA using DNase I (Takara, Japan), then reverse transcribed into cDNA using GoScript™ Reverse Transcription System (Promega, USA). RT-qPCR analysis was performed with a 1000 series Thermal Cycling Platform (Bio-Rad, USA) using EvaGreen 2×qPCR MasterMIX (Applied Biological Materials Inc.). At least three biological replicates and three technical replicates within an experiment for each sample were performed. The relative expression levels were calculated using the 2^−△△CT^ method. The *AT4G33380* was used as an internal control (Czechowski et al. 2005). All primers used are listed in Table S6.
